# Anti-inflammatory effects of ciprofloxacin in *S. aureus Newman *induced nasal inflammation *in vitro*

**DOI:** 10.1186/1476-9255-5-11

**Published:** 2008-07-29

**Authors:** F Sachse, C von Eiff, K Becker, C Rudack

**Affiliations:** 1 Department of Otorhinolaryngology Head and Neck Surgery, University of Muenster, Germany; 2 Department of Microbiology, University of Muenster, Germany

## Abstract

**Objectives:**

Chronic rhinosinusitis (CRS) is a chronic inflammatory disease of the nasal mucosa. Recent studies suggest that *S. aureus *enterotoxins may play an etiologic role in the development of CRS. Apart from surgery and repeated courses of steroids, macrolide antibiotics have been reported to exert anti-inflammatory effects in CRS. Similar effects have been reported for fluoroquinolones on various cell types. Since these effects have poorly been characterized in CRS, we examined anti-inflammatory effects of ciprofloxacin on human nasal epithelial cells (HNECs).

**Methods:**

Inflammation was induced in HNECs cultured from nasal turbinate mucosa with supernatants of *S. aureus Newman *for 12 hours. Subsequently, HNECs were coincubated with *S. aureus Newman *and ciprofloxacin (1.5 × 10^-5 ^M), clarithromycin (10^-6 ^M) or prednisolone (10^-5 ^M) for another 12 hours. IL-8 synthesis was quantified after 12 and 24 hours by ELISA.

**Results:**

Stimulation with *S. aureus Newman *supernatants was associated with an increase of IL-8 synthesis after 12 hours in all experiments. During the second 12 hours, IL-8 synthesis decreased and this effect was independent from any stimulus or inhibitor. However, coincubation of HNECs with ciprofloxacin was associated with a more extensive decrease of IL-8 synthesis. Similarly, addition of clarithromycin was associated with a reduction of IL-8 synthesis although this effect was not significant. Coincubation with prednisolone resulted in a significant reduction of IL-8 levels.

**Conclusion:**

Ciprofloxacin exerts anti-inflammatory effects in *S. aureus Newman *driven nasal inflammation. Inhibitory effects were comparable to those of prednisolone and clarithromycin.

## Introduction

Chronic rhinosinusitis (CRS) is an inflammatory disease of the nasal and paranasal mucosa that can broadly be classified in two major forms depending on the presence (CRSwNP) or absence of nasal polyps (CRSsNP) [1]. Recent studies suggest that *S. aureus *enterotoxins may play an etiologic role in the development of CRSwNP [2]. Therapy of CRSwNP often consists of repeated surgical reduction of nasal polyps and application of steroids. However, application of topical steroids even over several months often cannot prevent disease reoccurrence especially in CRSwNP. This may be due to the unknown underlying chronic inflammatory process and the phenomenon of steroid resistance that has also been demonstrated for nasal mucosa [3]. Consequently, alternative anti-inflammatory drugs that possess the potential to influence the chronic inflammatory process in CRSwNP are warranted.

Macrolide antibiotics exert anti-inflammatory effects and were first observed in patients with diffuse panbronchiolitis. In these patients a tremendous improvement of the 5 year survival rate was observed following treatment with macrolides [4]. The anti-inflammatory effects of macrolides have been explained by their anti-inflammatory capabilities which are independent from the antibiotic effect. These effects have also been studied in human nasal mucosa and human nasal epithelial cells (HNECs) for more than 10 years [5,6]. In addition, a double-blind, randomized, placebo-controlled trial on CRS patients treated with a low dose of roxythromycin, demonstrated clinical benefit particularly for CRS patients with low levels of IgE [7].

Fluoroquinolones such as ciprofloxacin are known as effective antibiotics with excellent activities against numerous respiratory tract pathogens. They are applied for the topical treatment of recurrent purulent otitis media, a common and often bacterial driven inflammatory disease of the middle ear. Moreover, similar to macrolides, fluoroquinolones have been reported to exert immunomodulatory effects on various cell types [8]. Since these effects have poorly been characterized in CRS, we examined the effects of ciprofloxacin on the IL-8 synthesis of HNECs stimulated with supernatants of *S. aureus Newman*. Thus, the antibiotic effect of ciprofloxacin was negligible since bacterial supernatants were used for stimulation but not bacteria. Strain *Newman *was chosen since it has been shown to produce enterotoxins which have recently been discussed to play a role in CRSwNP [2]. For comparative reasons, inhibition experiments with prednisolone and clarithromycin were included.

## Materials and methods

Unless declared otherwise, all reagents were purchased from Sigma (Deisenhofen, Germany).

Inferior turbinate mucosa was gained from two sinus healthy subjects with no signs of CRS (mean age 42 years) undergoing septoplasty/septorhinoplasty during routine surgery at the Department of Otorhinolaryngology of the University Hospital of Münster, Germany. None of the patients had undergone sinus surgery previously or received medical treatment four weeks prior to surgery. Allergy was excluded by skin-prick test and by RAST. Informed consent was obtained from all patients and the study was approved by the ethics committee of the University of Münster. In addition, the authors declare that they have no competing interests.

### Cell culture of HNECs

About 0.1–0.2 g wet tissue obtained from the inferior turbinate was dissected for the approach to individual experiments. Subsequently, tissue was washed with phosphate-buffered saline (PBS) and incubated with trypsin (0.5%) overnight at 4°C. The epithelial layer was then re-suspended in PBS (pH 7.4). After centrifugation at 1.000 g for 10 minutes, the cells were washed again with PBS, pelleted and re-suspended in a serum-free keratinocyte basal medium (Airway epithelial cell growth medium, AECG Medium, Promocell, Heidelberg, Germany), supplemented with a ready-to addition supplement according to the manufacturer's recommendation. Penicillin was applied to a dilution of 200 units/ml medium and streptomycin applied to a dilution of 0.2 μg/ml medium (Biochem, Berlin, Germany). Cells were grown to 80% confluence and passaged two more times. HNECs were then split and grown in 12 well plates (each well containing about 1,8 × 10^5 ^cells for a single experiment) to 80% confluence. The medium was exchanged by serum-free medium 24 hours prior to stimulation. The epithelial phenotype of cells was confirmed by staining of epithelial cells with a monoclonal anti-pan cytokeratin antibody as previously shown [9].

### Stimulation of HNECs

Viability of HNECs as assessed by trypan blue dye exclusion was greater than 95% in all experiments before and after stimulation.

Experiments were started by performing *S. aureus *induced inflammation in HNECs using *S. aureus Newman *D2C (ATCC 2590, dilution of 1:10) supernatants. After 12 hours culture supernatants were collected and IL-8 synthesis was quantified by ELISA in double determination according to the manufacturer's instructions (IL-8 protein detection range > 3.5 pg/ml, R&D, Wiesbaden, Germany). Subsequently, serum-free medium was added and HNECs were coincubated with *S. aureus Newman *supernatants and ciprofloxacin (1.5 × 10^-5 ^M, Bayer, Leverkusen, Germany), clarithromycin (10^-6 ^M, Abbot, Wiesbaden, Germany) or prednisolone (10^-5 ^M, Merck, Darmstadt, Germany) for another 12 hours. Again IL-8 levels were quantified by ELISA.

### Concentrations of inhibors

A dose-response relationship for prednisolone was tested in previous studies and similar to Wallwork et al. we found optimal inhibition of IL-8 levels at a concentration of 10^-5 ^M prednisolone [6,10].

For clarithromycin the concentration of 10^-6 ^M was based on serum levels of macrolides reached in paranasal sinus mucosa [11]. In addition, previous studies demonstrated decreased IL-8 synthesis by HNECs coincubated with LPS and erythromycin at a concentration of 10^-6 ^M [5]

Ciprofloxacin was used at a concentration of 1.5 × 10^-5 ^M. This concentration represents the concentration reached in tissues and was applied by Ulrich et al. in a study using primary human epithelial cells derived from nasal polyps [12,13].

Viability of cells was assessed by trypan blue exclusion test after 24 hours. Cell viability was greater than 95% in all experiments.

### Statistics

Two-way analysis of variance (ANOVA) was used to calculate differences in IL-8 synthesis as a result of stimulation with *S. aureus Newman*. Results were displayed as mean ± standard deviation. Values of *P *< .05 were considered to be significant.

## Results

**Figure 1:**
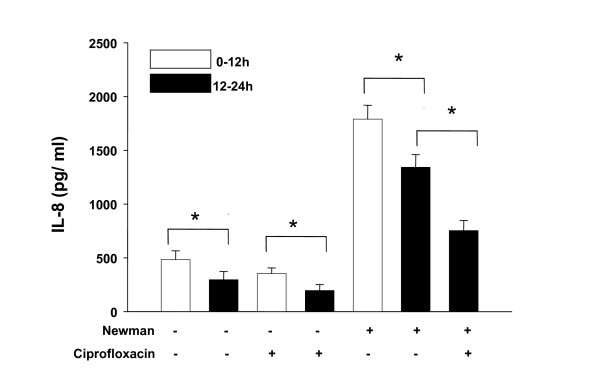
**IL-8 synthesis (pg/ml) following coincubation of HNECs with *S. aureus *supernatants and ciprofloxacin (1.5 × 10^-5 ^M)**. Bars represent means ± standard deviation of three independent experiments. Addition or absence of *S. aureus Newman *supernatants and ciprofloxacin is indicated by "+" and "-". p < 0.05 (*) was considered significant.

**Figure 2:**
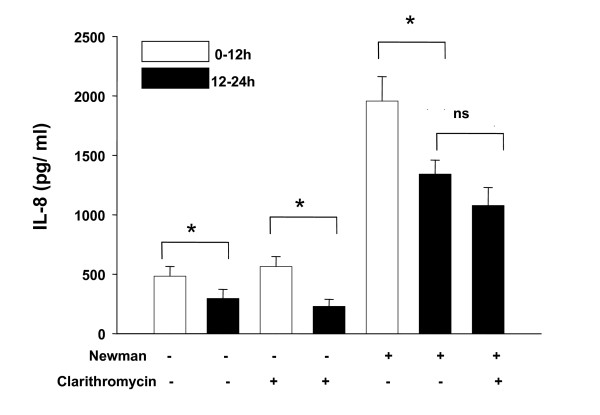
**IL-8 synthesis (pg/ml) following coincubation of HNECs with *S. aureus *supernatants and clarithromycin (10^-6 ^M)**. Bars represent means ± standard deviation of three independent experiments. Addition or absence of *S. aureus Newman *supernatants and clarithromycin is indicated by "+" and "-". p < 0.05 (*) was considered significant.

**Figure 3:**
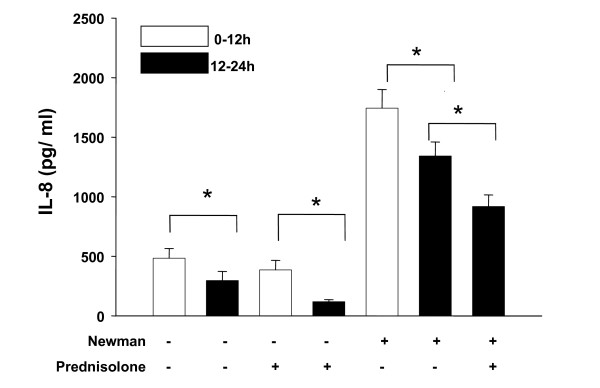
**IL-8 synthesis (pg/ml) following coincubation of HNECs with *S. aureus *supernatants and prednisolone (10^-5 ^M)**. Bars represent means ± standard deviation of three independent experiments. Addition or absence of *S. aureus Newman *supernatants and prednisolone is indicated by "+" and "-". p < 0.05 (*) was considered significant.

Addition of *S. aureus Newman *supernatants was associated with an increase of IL-8 synthesis after 12 hours in all experiments. However, we observed a significant decrease of IL-8 synthesis in controls and in controls of antibiotics and prednisolone that occurred during the second 12 hours (12–24 hours). Moreover, stimulation with *S. aureus Newman *alone was associated with an identical effect: the maximum of IL-8 synthesis was determined after 12 hours, whereas further stimulation with *S. aureus Newman *demonstrated decrease of IL-8 synthesis during the second 12 hours (12–24 hours). Taken together these results suggest that a time-dependent decrease of IL-8 synthesis occurred in HNECs which was independent from any stimulus or inhibitor. As a consequence, the time-dependent decrease of IL-8 synthesis had to be considered when analyzing inhibitory effects of ciprofloxacin, clarithromycin and prednisolone (fig. 1, 2, 3).

We found that coincubation of HNECs with ciprofloxacin (1.5 × 10^-5 ^M) resulted in a significant decrease of IL-8 synthesis (p < 0.05). Similarly, addition of clarithromycin (10^-6 ^M) was associated with a reduction of IL-8 synthesis although this effect was not significant (p > 0.05). Coincubation of HNECs with supernatants of *S. aureus Newman *and prednisolone (10^-5 ^M) resulted in a significant reduction of IL-8 levels (p < 0.05) (fig. 1, 2, 3).

## Discussion

Macrolides have been studied for years and found to exert immunomodulatory effects that are independent from their antibiotic effects [4]. Although similar effects have been reported for the class of fluoroquinolones, their anti-inflammatory potential on the nasal epithelium has poorly been characterized [8].

In this study we analyzed the IL-8 response by HNECs coincubated with *S. aureus Newman *and ciprofloxacin. In a previous series of experiments we had already quantified Eotaxin which is a chemoattractant for eosinophils in nasal polyps. However, an induction of this chemokine in HNECs following stimulation with *S. aureus *supernatants was not observed.

Since HNECs were stimulated with bacterial supernatants but not with bacteria, any inhibition of the IL-8 response by ciprofloxacin could be attributed to immunomodulatory effects. We observed that ciprofloxacin significantly decreased IL-8 synthesis by HNECs. Moreover, inflammation was induced by *S. aureus *supernatants in our study and strain *Newman *has been shown to produce *S. aureus enterotoxin a (sea) *[14]. This is worth mentioning since *S. aureus *enterotoxins have been considered to play a role in CRSwNP [2].

Ciprofloxacin belongs to the fluoroquinolones and displays excellent activities against numerous respiratory tract pathogens. It is known to strongly accumulate in human neutrophils and to easily penetrate epithelial cells. Moreover it has been demonstrated to exert protective effects against H. influenzae and Pseudomonas aeruginosa in nasal polyp epithelial cells. In addition, infected cells could be rescued at a higher rate if ciprofloxacin was added to the culture [13].

Apart from these bactericidal effects, immunomodulatory effects of fluoroquinolones have been reported. However, in vitro exposure of various cells to fluoroquinolones alone did not exert any measurable immunomodulatory effect. Stimulation or inhibition of cytokine synthesis has only been observed when cells were exposed to a stimulant or stress [8]. In HNECs derived from human nasal polyps levofloxacin was found to down-regulate the synthesis of pro-inflammatory cytokines TNF-α, IFN-γ and IL-8 [15]. In stimulated airway epithelial cells it was found that grepafloxacin inhibited the TNF-α stimulated IL-8 synthesis.

Factors that have been associated to regulate the immunomodulatory activity of quinolones include effects on intracellular cyclic AMP (cAMP) and phosphodiesterases, transcription factors (NF-κB, AP-1, NF-IL-6, NFAT) and topoisomerase II interactions [8]. Taken together, quinolones exert their effects on the synthesis of cytokines through modulation of cellular transcription factors although the exact events are still unknown and mandates further studies.

For comparative reasons we also studied the effects of clarithromycin. Addition of 10^-6 ^M clarithromycin was associated with a decrease of IL-8 synthesis after 12 hours although inhibition failed to reach significance. However, previous studies did demonstrate decreased IL-8 synthesis by HNECs coincubated with LPS and erythromycin even at a concentration of 10^-6 ^M [5]. Our intention to use a concentration of clarithromycin of 10^-6 ^M was based on based on serum levels reached in clinical practice [11]. On the other hand it has been observed that macrolides preferentially accumulate at sites of inflammation and therefore concentrations higher than plasma levels can be reached.

Apart from a lower concentration of 10^-6 ^M clarithromycin we incubated our cells for 12 hours, whereas in most other studies cells were incubated for 24 hours at a concentration of 10^-5 ^M clarithromycin [5,6]. Wallwork et al. who used cultures of whole sections of sinus mucosa from CRS patients found a reduction of IL-8 levels after 24 hours of incubation with 10^-5 ^M clarithromycin [6].

The anti-inflammatory effect of macrolides has been attributed to inhibition of the activation of NF-κB. Miyanohara et al. have evaluated the effects of clarithromycin on the H. influenzae endotoxin induced expression of IL-1β and ICAM-1 in HNECs and human nasal fibroblasts. He found that clarithromycin reduced DNA-binding activity of NF-κB in HNECs and human nasal fibroblasts as demonstrated by EMSA [16]. Clinical relevance has been recently reported by Wallwork et al. who demonstrated clinical benefit and decrease of IL-8 levels particularly in a subgroup of CRS patients with low levels of IgE that had been treated with erythromycin [7].

For comparative reasons we included experiments with prednisolone since it has been well established that steroids are capable to inhibit inflammatory reactions by the reduction of cytokines such as IL-8 [17]. We found decreased IL-8 synthesis by HNECs following coincubation with *Newman *supernatants and prednisolone. However, failure of IL-8 reduction by prednisolone has been reported in CRS, too [18].

## Conclusion

Taken together, we demonstrated anti-inflammatory effects of ciprofloxacin on the IL-8 synthesis in *S. aureus Newman *driven nasal inflammation in vitro. These anti-inflammatory effects were comparable to those of prednisolone and clarithromycin. Fluoroquinolones may therefore represent an alternative target to reduce inflammatory mediators in the nasal mucosa. However, further studies are necessary for a detailed understanding of the immunomodulatory potential of fluoroquinolones.

## Competing interests

The authors declare that they have no competing interests.

## Authors' contributions

FS designed and performed the study. Culturing and stimulation of human nasal epithelial cells as well as evaluating experimental data was done by FS. CR designed the study, quantified IL-8 levels using ELISA and was involved with interpretation of results. CvE and KB prepared and characterized *S. aureus Newman *supernatants used in this study. All authors read and approved the final manuscript.
